# Shoulder Arthrodesis versus Upper Trapezius Transfer for Traumatic Brachial Plexus Injury: A Proportional Meta-Analysis

**DOI:** 10.1155/2021/4445498

**Published:** 2021-10-13

**Authors:** Dafang Zhang, Rohit Garg, Brandon E. Earp, Philip Blazar, George S. M. Dyer

**Affiliations:** ^1^Department of Orthopaedic Surgery, Brigham and Women's Hospital, 15 Francis St, Boston, MA 02115, USA; ^2^Department of Orthopaedic Surgery, Massachusetts General Hospital, 55 Fruit St, Boston, MA 02114, USA; ^3^Harvard Medical School, 25 Shattuck St, Boston, MA 02115, USA

## Abstract

Shoulder arthrodesis and upper trapezius transfer are two surgical options for secondary shoulder reconstruction for traumatic brachial plexus injury (BPI). There is a lack of comparative evidence to guide the choice for one procedure over the other. The objectives of this study were to compare (1) rates of complications and reoperation and (2) shoulder range of motion and functional outcome scores following shoulder arthrodesis versus upper trapezius transfer for traumatic BPI. A systematic review and meta-analysis were conducted by a search of four databases of studies assessing shoulder arthrodesis and/or upper trapezius transfer for shoulder reconstruction following adult traumatic BPI. A proportional meta-analysis was performed using a random effects model in anticipation of unobserved heterogeneity. The final meta-analysis included 374 patients from 17 studies, including 232 patients from 11 studies on shoulder arthrodesis and 142 patients from 6 studies on upper trapezius transfer. Shoulder arthrodesis had higher rates of complications and reoperations than upper trapezius transfer for traumatic BPI, but these differences did not reach a statistical significance. Due to the limited sample size, variations in reporting, and study heterogeneity in the published literature, we were not able to draw conclusions regarding shoulder range of motion and functional outcome scores between these two procedures. Shoulder arthrodesis and upper trapezius transfer are both viable options for secondary shoulder reconstruction for traumatic BPI, but with different complications and reoperation profiles. Patients should be counseled on the risk of nonunion and humerus fracture following shoulder arthrodesis.

## 1. Introduction

Adult traumatic brachial plexus injuries (BPI) are uncommon but devastating injuries to the upper extremity [1]. The primary reconstruction of traumatic BPI generally consists of nerve grafting, nerve transfer, or tendon transfer. Lack of shoulder function is a common problem after traumatic BPI due to paralysis of the deltoid, supraspinatus, and infraspinatus. Such a flail shoulder can arise due to the severity of the initial injury, failure of spontaneous recovery, paucity of viable donor nerves for primary nerve reconstruction, or failure of primary nerve reconstruction [2]. A flail shoulder can cause pain due to inferior instability of the glenohumeral joint, and an unstable glenohumeral joint can adversely affect function by the unwanted dissipation of elbow flexion power [3].

Secondary reconstruction options for the flail shoulder in the setting of traumatic BPI include shoulder arthrodesis [3] and upper trapezius transfer [2, 4]. Intact motor function in the periscapular muscles, including the trapezius, levator scapulae, serratus anterior, and rhomboids, is preferred for an optimal functional result after shoulder arthrodesis, but not required [3]. The prerequisites for upper trapezius transfer include at least M4 strength in the trapezius muscle, passive glenohumeral joint motion to at least 80°, and no advanced degeneration of the glenohumeral joint [5–8]. There is a lack of comparative evidence of shoulder arthrodesis versus upper trapezius transfer for the secondary reconstruction of traumatic BPI to provide patients and surgeons guidance in choosing between these two surgical options.

The objectives of this study were to perform a systematic review and proportional meta-analysis of the available published data to determine whether there are any differences in (1) postoperative complications and reoperations and (2) shoulder range of motion and functional outcome scores after shoulder arthrodesis versus upper trapezius transfer for the secondary reconstruction of traumatic BPI. Our null hypotheses were that no differences in the rate of complication, the rate of reoperation, final range of motion, and functional outcome scores exist between these two procedures.

## 2. Materials and Methods

A systematic review and meta-analysis were conducted according to the Cochrane Handbook for Systematic Reviews of Interventions guideline and reported according to the Preferred Reporting Items for Systematic Reviews and Meta-Analyses (PRISMA) guideline.

### 2.1. Types of Studies

Randomized and nonrandomized comparative studies and observational studies were considered for inclusion. Editorials, letters, commentaries, protocols, practice guidelines, systematic reviews, and animal studies were not included. There were no language or publication date criteria for inclusion.

### 2.2. Types of Interventions

Studies assessing clinical outcomes of shoulder arthrodesis and/or upper trapezius transfer for shoulder reconstruction following adult traumatic BPI were considered for inclusion. We excluded studies of interventions pertaining to brachial plexus birth palsy, iatrogenic BPI, nontraumatic BPI, radiation-induced BPI, isolated axillary nerve palsy, and other pathologies for which shoulder arthrodesis and/or upper trapezius transfer may be performed, such as poliomyelitis. Studies of combined indications were excluded if greater than 10% of patients underwent shoulder arthrodesis and/or upper trapezius transfer for an indication other than traumatic BPI.

### 2.3. Search Strategy

A comprehensive search of the earliest available publications to September 20, 2020, was conducted of four databases: MEDLINE, EMBASE, CENTRAL, and the U.S. National Institutes of Health registry of clinical trials (https://www.ClinicalTrials.gov). MeSH headings and subheadings were used in various combinations and supplemented with free text to optimize sensitivity. Keywords related to the pathology (e.g., “brachial plexus”) were combined with keywords related to the study interventions (e.g., “shoulder arthrodesis” and “trapezius transfer”). The bibliographies of pertinent studies were reviewed for additional studies meeting inclusion criteria.

### 2.4. Outcome Measures

The main outcome measures of this meta-analysis were (1) postoperative complications and reoperations and (2) shoulder range of motion and functional outcome scores at the time of final follow-up.

### 2.5. Data Extraction

Titles and abstracts of candidate studies were screened according to inclusion and exclusion criteria, and studies were excluded if they did not pertain to the topic under review. Subsequently, the full-text articles of the remaining potentially eligible studies were retrieved and reviewed for data extraction versus exclusion. Data extraction was performed using a standardized data extraction form, which included study demographics, study interventions, follow-up duration, and outcomes.

### 2.6. Risk of Bias

All 17 included studies in this systematic review were observational studies (level IV therapeutic evidence). The Methodological Index for Nonrandomized Studies (MINORS) score was used to assess the quality of each study and the potential for bias [9].

### 2.7. Statistical Analysis

Since the systematic review yielded observational studies of shoulder arthrodesis and/or upper trapezius transfer, not comparative studies, a proportional meta-analysis was performed [10]. A meta-analysis was performed for the prevalence of postoperative complications and reoperations using a random effects model in anticipation of unobserved heterogeneity. Similarly, a meta-analysis of range of motion was performed using a random effects model. Since the observational studies in the meta-analysis are heterogeneous (e.g., age, BPI pattern, surgical technique, and rehabilitation), a random effects model was chosen and assumes that the true effect varies between studies. The combined effect in a random effects model estimates the mean effect in a distribution of effects assumed to be a random sample. Weights are assigned to studies in a random effects model by the inverse of the variance of the effect, taking into account within-study and between-study variances. Compared with a fixed effects model, weights assigned in a random effects model are generally more balanced, such that larger studies are not overly dominant and small studies are not ignored. Heterogeneity was measured using the *I*^2^ statistic. The standard significance criteria of *p* < 0.05 was used. Meta-analysis was performed using MetaXL version 5.3 for Windows (Brisbane, Queensland, Australia).

### 2.8. Study Characteristics and Quality

**Figure 1:**
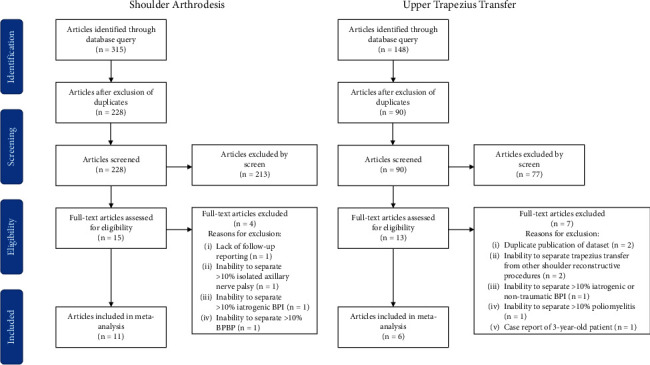
Flowchart illustrating the article selection and exclusion process for the systematic review and meta-analysis.

Four hundred sixty-three articles were identified by the initial query, including 315 articles by querying shoulder arthrodesis and 148 articles by querying trapezius transfer. After exclusion of duplicate articles, 318 articles remained; these articles were screened according to our inclusion and exclusion criteria. Twenty-eight full-text articles were reviewed, and after application of inclusion and exclusion criteria, 17 articles were included in the final meta-analysis, including 11 articles on shoulder arthrodesis and 6 articles on upper trapezius transfer (Figure 1) [5, 8, 11–23].

The meta-analysis included 374 patients, with a mean age of 29 years, 92% of whom were male. The mean duration of time from injury to surgery was 46 months. The mean duration of postoperative follow-up was 38 months, and minimum postoperative follow-up was 6 months (Table 1). The majority of shoulder arthrodeses was performed for complete BPI, whereas the plurality of upper trapezius transfers was performed for upper trunk BPI (Table 2).

The meta-analysis included 232 patients who underwent shoulder arthrodesis at a mean age of 28 years, 94% of whom were male. The mean duration of time from injury to shoulder arthrodesis was 37 months, and the mean duration of postoperative follow-up was 48 months. The meta-analysis included 142 patients who underwent upper trapezius transfer at a mean age of 29 years, 88% of whom were male. The mean duration of time from injury to upper trapezius transfer was 54 months, and the mean duration of postoperative follow-up was 22 months.

All 17 studies included in this meta-analysis were retrospective observational studies, with a mean MINORS score of 9.8 (range 9-10). As the global ideal MINORS score is 16 for noncomparative studies, this is indicative of a moderate risk of bias in the included studies [25]. All included studies had a minimum of 6 months postoperative follow-up, and 16 out of 17 had a minimum of 1 year postoperative follow-up. Only 9 out of 17 studies specified the pattern of brachial plexus injury. No study reported an adequate discussion of power.

## 3. Results

### 3.1. Complications and Reoperations

**Figure 2:**
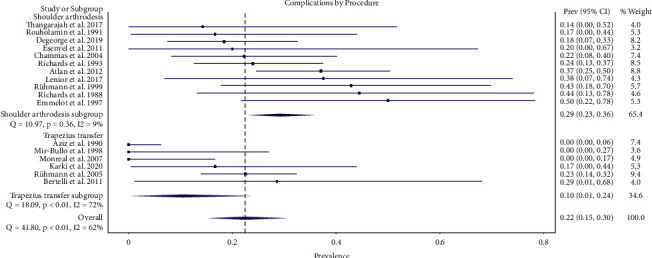
Forest plot illustrating the proportions of complications for shoulder arthrodesis versus upper trapezius transfer.

Eighty-nine complications occurred in 374 procedures (24%) in this meta-analysis. Sixty-seven complications occurred in 232 shoulder arthrodesis procedures (29%), and 22 complications occurred in 142 upper trapezius transfer procedures (15%) (Table 3). The overall weighted prevalence of complication was 0.22 (95% CI 0.15–0.30). The weighted prevalence of complications in the shoulder arthrodesis group was 0.29 (95% CI 0.23–0.36). The weighted prevalence of complications in the upper trapezius transfer group was 0.10 (95% CI 0.01–0.24). The difference in the complication rate between the two procedures was not statistically significant (Figure 2).

**Figure 3:**
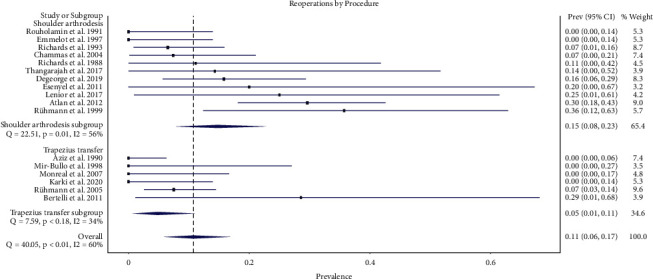
Forest plot illustrating the proportions of reoperations for shoulder arthrodesis versus upper trapezius transfer.

Forty-five reoperations (12%) were performed in this meta-analysis. Thirty-seven reoperations were performed in the shoulder arthrodesis group (16%), and 8 reoperations were performed in the upper trapezius transfer group (6%) (Table 4). The overall weighted prevalence of reoperation was 0.11 (95% CI 0.06–0.17). The weighted prevalence of reoperations in the shoulder arthrodesis group was 0.15 (95% CI 0.08–0.23). The weighted prevalence of reoperations in the upper trapezius transfer group was 0.05 (95% CI 0.01–0.11). The difference in the complication rate between the two procedures was not statistically significant (Figure 3).

**Figure 4:**
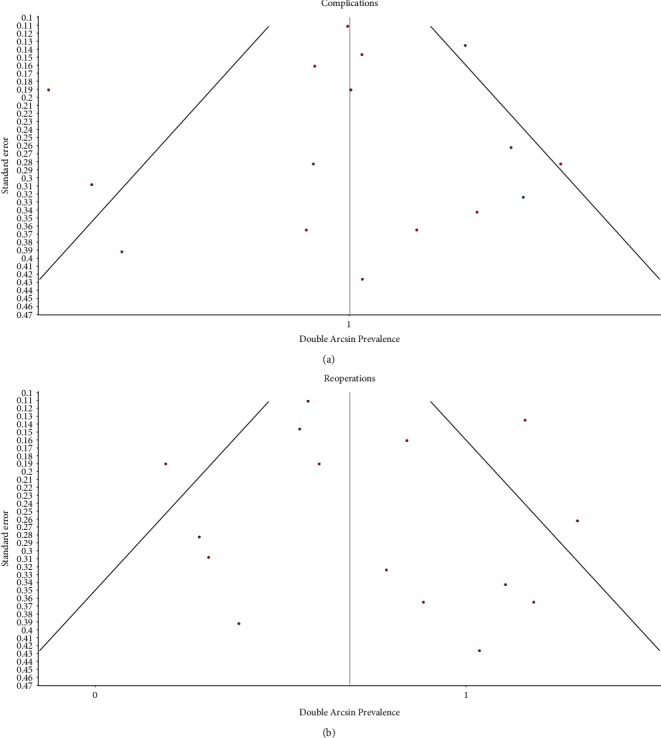
Funnel plots of the meta-analysis of data on (a) complications and (b) reoperations after shoulder arthrodesis and upper trapezius transfer for traumatic BPI, illustrating low publication bias. The area within the funnel represents where 95% of data points would be expected in the absence of publication bias.

There was substantial heterogeneity among studies in the proportional meta-analysis of complications (*I*^2^ = 62%) and reoperations (*I*^2^ = 60%). [26] Funnel plots of the proportional meta-analysis of data on complications and reoperations do not demonstrate asymmetry to suggest publication bias (Figure 4).

### 3.2. Range of Motion and Functional Outcome Scores

**Figure 5:**
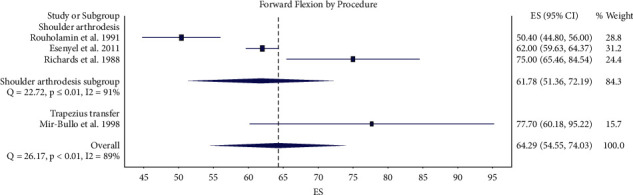
Forest plot illustrating active shoulder forward flexion for shoulder arthrodesis versus upper trapezius transfer.

**Figure 6:**
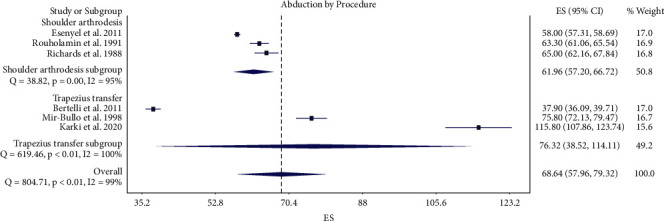
Forest plot illustrating active shoulder abduction for shoulder arthrodesis versus upper trapezius transfer.

Three studies in the shoulder arthrodesis group [11, 20, 21] and one study in the upper trapezius transfer group [15] reported final postoperative active shoulder forward flexion. The pooled final postoperative active shoulder forward flexion was 62° (95% CI 51–72°) in the shoulder arthrodesis group and 78° (95% CI 60–95°) in the upper trapezius transfer group, not a statistically significant difference (Figure 5). Three studies in the shoulder arthrodesis group [16, 21, 22] and three studies in the upper trapezius transfer group [5, 6, 17] reported final postoperative active shoulder abduction. The pooled final postoperative active shoulder abduction was 62° (95% CI 57–67°) in the shoulder arthrodesis group and 76° (95% CI 39–114°) in the upper trapezius transfer group, not a statistically significant difference (Figure 6). There was considerable heterogeneity among studies in the meta-analysis of forward flexion (*I*^2^ = 89%) and abduction (*I*^2^ = 99%) [26].

Three studies in the shoulder arthrodesis group [11, 16, 23] and one study in the upper trapezius transfer group [17] reported functional outcome scores. Atlan et al. reported a mean Disabilities of the Arm, Shoulder, and Hand (DASH) Score of 35.6 and American Shoulder and Elbow Surgeons (ASES) Shoulder Score of 69 in 54 patients who underwent shoulder arthrodesis with mean 37-month follow-up [11]. Esenyel et al. reported on a mean Oxford Shoulder Score (OSS) of 35 in 5 patients who underwent shoulder arthrodesis with mean 58-month follow-up [16]. Thangarajah et al. reported a mean OSS of 27 and subjective shoulder value (SSV) of 45 in 7 patients who underwent shoulder arthrodesis with mean 98-month follow-up [23]. Karki et al. reported a mean DASH score of 38 in 12 patients who underwent upper trapezius transfer with mean 24-month follow-up [17].

## 4. Discussion

Secondary shoulder reconstruction is commonly required in the treatment of adult traumatic BPI. The need for secondary shoulder reconstruction can arise in a variety of clinical scenarios, ranging from complete BPI with a paucity of donor options for shoulder reanimation to the failure of primary nerve reconstruction. Shoulder arthrodesis and upper trapezius transfer are two surgical options for secondary shoulder reconstruction for traumatic BPI. While there are differences in the indications for these two procedures [3, 5, 7, 8], most traumatic BPI patients are candidates for both, and there is no comparative evidence to guide the choice for one procedure over the other. The goal of the present study was to synthesize the available literature on these procedures and assess for differences in complication rates, reoperation rates, shoulder range of motion, and functional outcome scores in patients with traumatic BPI. Due to the heterogeneity of the studies in our meta-analysis, a random effects model was used, which assigned study weights accounting for within-study and between-study variances. With the current available evidence, we found higher rates of complication and reoperation after shoulder arthrodesis compared with upper trapezius transfer for traumatic BPI, but this effect did not reach the statistical significance. Due to the limited sample size, variations in reporting, and study heterogeneity in the published literature, we were not able to draw conclusions regarding differences in shoulder range of motion and functional outcome scores between these two procedures.

This study has several limitations. First, the findings of our meta-analysis are limited by the available literature and the quality of the included studies. Since no comparative studies between shoulder arthrodesis and upper trapezius transfer were identified, a proportional meta-analysis was performed [10]. The included studies had a moderate risk of bias according to the MINORS score [26]. Second, there was substantial heterogeneity in the included studies, in terms of injury severity and pattern, surgical technique and postoperative care, and outcomes reporting. There was a substantial to considerable amount of heterogeneity in the results of our meta-analysis according to the Cochrane Handbook for Systematic Reviews of Interventions guidelines [12]. The large amount of study heterogeneity limited the conclusions that we were able to draw from this study. Third, since this meta-analysis included all adult traumatic BPI, we were unable to substratify our analyses to assess each BPI pattern, such as upper trunk BPI, extended upper trunk BPI, or global BPI. It is possible that some BPI patterns benefit more from one shoulder reconstructive option than another. Finally, there is the potential for publication bias, especially due to the inclusion of smaller case series. If favorable outcomes of surgical interventions were preferentially published in the literature, then our meta-analysis may underestimate the true rates of complications and reoperations and overestimate the true expected shoulder motion and functional outcomes after these procedures.

Shoulder arthrodesis demonstrated higher rates of complications and reoperations compared with upper trapezius transfer for traumatic BPI. It is possible that with greater power, these differences in operative complications and the need for future surgery may have reached a statistical significance. While the difference in these dichotomous outcomes was not statistically significant in our analysis, it is important to note that the reported complications following shoulder arthrodesis tended to be more severe and more frequently require reoperation than those following upper trapezius transfer. Surgeons should be aware of the complications and reoperation profiles of each procedure. Common complications unique to shoulder arthrodesis are nonunion of the arthrodesis and fracture of the humeral shaft distal to arthrodesis. The rate of nonunion of shoulder arthrodesis was 9% in our meta-analysis, but 100% of these required reoperations. Humeral shaft fractures following shoulder arthrodesis arise from stress risers in the bone distal to a stiff construct; in the reported literature, these have been treated successfully both operatively and nonoperatively [11, 13, 16, 20]. Upper trapezius transfers are largely successful for improving painful inferior instability from a previously flail shoulder; only 1% of upper trapezius transfer cases in our meta-analysis underwent subsequent shoulder arthrodesis for persistent glenohumeral instability [24].

We were not able to draw conclusions regarding differences in shoulder range of motion and functional outcome scores between shoulder arthrodesis and upper trapezius transfer for traumatic BPI. Functional outcome scores were usually not reported, and when they were, an array of difference scoring systems was used, including DASH, OSS, ASES, and SSV, making direct comparisons challenging. Similarly, postoperative shoulder range of motion was inconsistently reported among the studies in this meta-analysis. There was no consensus about reporting forward flexion, abduction, external rotation, or a combination thereof. Some studies reported only postoperative motion, while others also reported preoperative motion, and still, others reported change in motion. Some studies reported abduction as the angle measured between the trunk and the arm [5, 7], while others utilized preset hand excursion parameters, such as the ability to reach the mouth, the forehead, and the buttock [18]. It is important to note that intact scapulothoracic motion portends a better prognosis for motion after both shoulder arthrodesis and upper trapezius transfer [3]. In our meta-analysis, there was a higher proportion of complete BPI in the shoulder arthrodesis group; worse scapulothoracic motion in this group may have biased our findings against shoulder arthrodesis. Prospective and comparative studies are needed to evaluate for differences in motion and functional outcomes after these two procedures in traumatic BPI.

## 5. Conclusions

Shoulder arthrodesis and upper trapezius transfer are both viable options for secondary shoulder reconstruction in adult traumatic BPI. Shoulder arthrodesis carries higher rates of complications and reoperations compared with upper trapezius transfer, but the differences are not statistically significant. Patients and surgeons should be aware of different complications and reoperation profiles of these two procedures. In particular, patients should be counseled on the risk of nonunion and humerus fracture with shoulder arthrodesis. Future comparative studies are necessary to clarify the difference in expected functional outcomes and shoulder motion after these two procedures in traumatic BPI.

## Figures and Tables

**Table 1:** Baseline study and patient characteristics^*∗*^.

Study	*n*	Male (*n*)	Mean age (years)	Delay to surgery (months)	Follow-up (months)
Shoulder arthrodesis					
Richards et al. [21]	9	8	27	56	25
Rouholamin et al. [22]	12	11	29	NR	34
Richards et al. [20]	46	44	30	NR	44
Emmelot et al. [15]	12	10	19	32	84
Rühmann et al. [8]	14	12	34	47	14
Chammas et al. [13]	27	25	25	33	71
Esenyel et al. [16]	5	5	40	NR	58
Atlan et al. [11]	54	54	24	31	37
Lenior et al. [18]	8	8	33	46	28
Thangarajah et al. [23]	7	6	48	60	98
Degeorge et al. [14]	38	NR	30	NR	58

Upper trapezius transfer					
Aziz et al. [12]	27	23	31	31	15
Mir-Bullo et al. [6]	6	6	28	30	19
Rühmann et al. [24]	80	69	31	73	29
Monreal et al. [7]	10	8	28	37	18
Bertelli et al. [5]	7	7	28	21	12
Karki et al. [17]	12	12	27	24	6

^*∗*^NR, not reported.

**Table 2:** Patterns of brachial plexus injury for the study cohort.

	C5	C5-C6	C5–C7	C5–C8	C8–T1	C5–T1	Total
Shoulder arthrodesis	1 (1%)	36 (24%)	28 (19%)	0 (0%)	2 (1%)	81 (55%)	148
Upper trapezius transfer	0 (0%)	26 (46%)	15 (27%)	4 (7%)	0 (0%)	11 (20%)	56
Total	1 (0.5%)	62 (30.4%)	43 (21.1%)	4 (2.0%)	2 (1.0%)	92 (45.0%)	204^*∗*^

^*∗*^Patterns of brachial plexus injury was available for 204 out of 374 patients in this meta-analysis.

**Table 3:** Complications following shoulder arthrodesis and upper trapezius transfer.

*n*	Complication
Shoulder arthrodesis	
22	Nonunion
15	Humerus fracture
11	Symptomatic hardware
5	Deep infection
5	Pin tract infection
2	Skin breakdown
1	Fixation failure
1	Hardware loosening
1	Hematoma
1	Intraarticular screw in acromioclavicular joint
1	Malunion
1	Scapular neck fracture
1	Superficial infection

Upper trapezius transfer	
7	Hardware loosening
4	Deep infection
2	Humerus fracture
2	Persistent glenohumeral joint instability
2	Skin breakdown
2	Transient musculocutaneous nerve palsy
1	Contralateral ulnar neuropathy
1	Silk suture extrusion
1	Superficial infection

**Table 4:** Reoperations following shoulder arthrodesis and upper trapezius transfer.

*n*	Reoperation
Shoulder arthrodesis	
23	Revision arthrodesis ± bone grafting for nonunion
7	Humeral shaft open reduction internal fixation
3	Irrigation, debridement, and hardware removal
2	Irrigation and debridement
1	Hematoma evacuation
1	Symptomatic hardware removal

Upper trapezius transfer	
4	Irrigation, debridement, and hardware removal
2	Shoulder arthrodesis
2	Symptomatic hardware removal

## Data Availability

The data used to support the findings of this study are available from the corresponding author upon request.

## Additional Points

Location of the study: the work for the study was carried out at Brigham and Women's Hospital.
